# Wide bandgap OPV polymers based on pyridinonedithiophene unit with efficiency >5%†
†Electronic supplementary information (ESI) available. See DOI: 10.1039/c5sc01427a



**DOI:** 10.1039/c5sc01427a

**Published:** 2015-06-04

**Authors:** Alexander M. Schneider, Luyao Lu, Eric F. Manley, Tianyue Zheng, Valerii Sharapov, Tao Xu, Tobin J. Marks, Lin X. Chen, Luping Yu

**Affiliations:** a Department of Chemistry and The James Franck Institute , The University of Chicago , 929 E 57th Street , Chicago , IL 60637 , USA . Email: lupingyu@uchicago.edu; b Department of Chemistry and The Argonne Northwestern Solar Energy Research Center , Northwestern University , 2145 Sheridan Road , Evanston , IL 60208 , USA . Email: l-chen@northwestern.edu; c Chemical Sciences and Engineering Division , Argonne National Laboratory , 9700 S. Cass Ave. , Lemont , IL 60439 , USA

## Abstract

We report the properties of a new series of wide band gap photovoltaic polymers based on the *N*-alkyl 2-pyridone dithiophene (**PDT**) unit. These polymers are effective bulk heterojunction solar cell materials when blended with phenyl-C_71_-butyric acid methyl ester (PC_71_BM). They achieve power conversion efficiencies (up to 5.33%) high for polymers having such large bandgaps, *ca.* 2.0 eV (optical) and 2.5 eV (electrochemical). Grazing incidence wide-angle X-ray scattering (GIWAXS) reveals strong correlations between π–π stacking distance and regularity, polymer backbone planarity, optical absorption maximum energy, and photovoltaic efficiency.

## Abstract 2

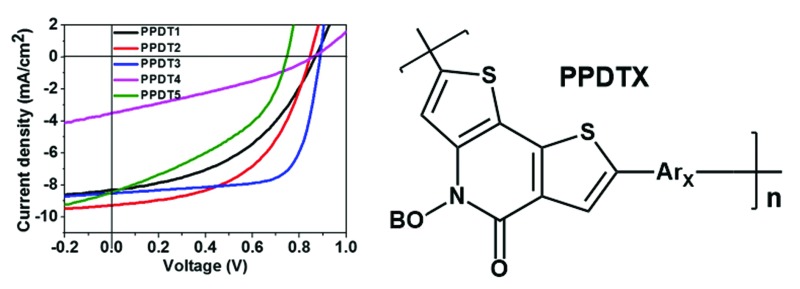
We report the properties of a new series of wide band gap photovoltaic polymers based on the *N*-alkyl 2-pyridone dithiophene (**PDT**) unit.

## Introduction

Notable recent progress has been made in research on organic solar cells (OSCs) in particular those based on polymers (PSCs), which show promise as a green technology to convert solar energy into electricity.^1^ This progress is driven by interdisciplinary research advances, ranging from the synthesis of novel materials,^2–11^ to innovative new device structures,^12–19^ and to better understanding of the device physics.^20–27^ This effort has culminated in devices with power conversion efficiencies (PCEs), the key OSC efficiency parameter, exceeding 10% in both single^6^ and tandem^19^ bulk heterojunction (BHJ) device architectures. In organic photovoltaic (OPV) devices, one of the critical challenges is to achieve optimal sunlight absorption in the active layer, especially in the near infrared region. From a materials engineering perspective, this can be accomplished by designing polymers that exhibit low bandgaps which extend light harvesting over a broad portion of the solar spectrum. Indeed, after extensive worldwide research effort, many low bandgap polymers have been developed, which have played an important role both in achieving high solar cell efficiencies and providing critical materials for device optimization research.

After initial research on moderate- to wide-bandgap polymers such as P3HT^28^ and PCDTBT,^29^ the community has recently focused extensively on the development of low bandgap polymers, with comparatively less focus on wide bandgap polymers, and especially the limited number having bandgaps near or above 2 eV which also exhibit PCEs greater than 5%.^30–33^ While wide bandgaps are a disadvantage for light absorption, such a deficiency might be compensated by attaining a higher open circuit voltage (*V*_oc_). More importantly, because almost all state-of-the-art high efficiency PSCs are fabricated with low bandgap polymers (absorption maxima between 600 to 700 nm) and PCBM, wide bandgap polymers (absorption maxima below 600 nm) provide opportunities to serve as additional donor materials in systems which feature two or more donor components due to the complementary nature of their absorption spectra. Such multiple-donor systems include the aforementioned tandem cells,^13,14,19^ but also the recently discovered ternary blend PSCs which have achieved high PCEs while maintaining simplicity of device fabrication, unlike tandem cells.^34–36^ The PCEs of state-of-the-art ternary blend PSCs have recently reached >8%.^37–40^

In this contribution, our efforts focus on designing and characterizing a new series of wide bandgap polymers which maintain large PCEs. We report here the development of one such system based on the *N*-alkyl 2-pyridone dithiophene (**PDT**) unit. This unit derives from our previous success with fused amide-linked systems to create the thieno[2′,3′:5′,6′]pyrido[3,4-*g*]thieno[3,2-*c*]isoquinoline-5,11(4*H*,10*H*)-dione (**TPTI**) monomer for use in polymeric acceptor systems.^41^ In comparison to **TPTI**, the **PDT** unit is relatively more electron-rich and thus proved useful as a component in donor polymer systems (Fig. S1†). A series of polymers are synthesized and characterized, which incorporate several comonomer motifs. Detailed analysis reveals that solar cells prepared from these materials and PC_71_BM exhibit a high *V*_oc_ of approximately 0.9 V and an optimized PCE as high as 5.33% in a conventional device structure. More importantly, due to their wide bandgap, yet deep HOMO levels, these polymers are expected to be useful components in tandem and ternary PSC systems. Future work in our group will focus on their use to that effect.

## Results and discussion

### Synthesis of monomers and polymers

**Fig. 1:**
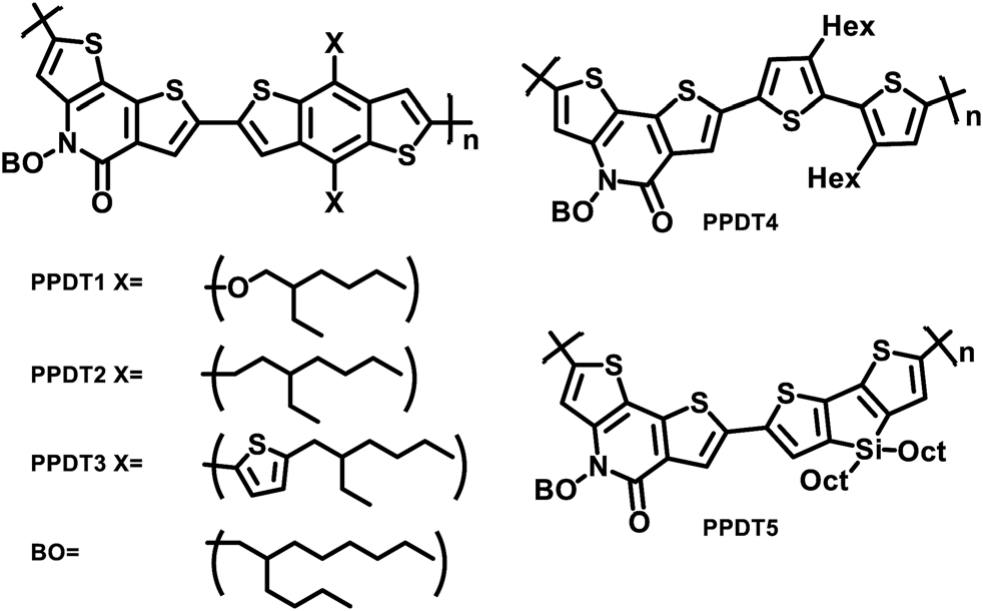
Chemical structures of **PPDT** series polymers.

**Scheme 1:**
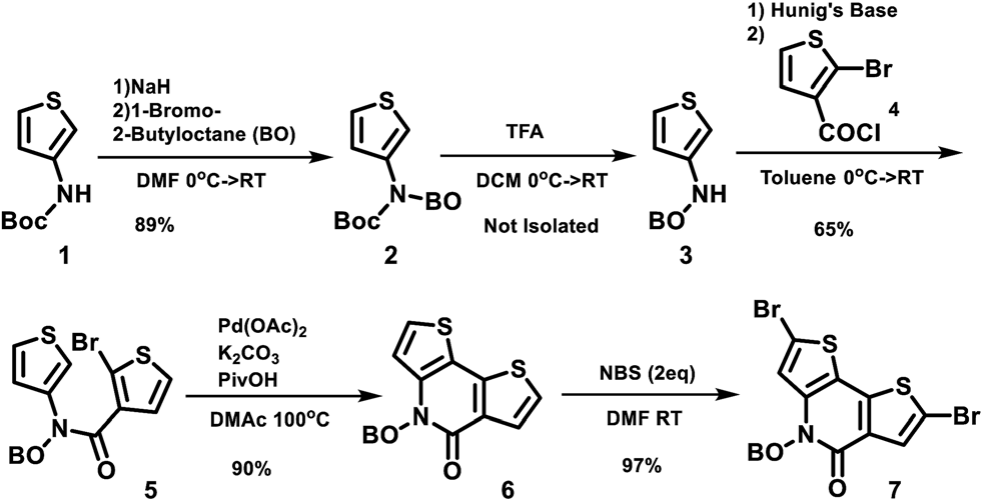
Synthetic scheme for **PDT** units.

**Scheme 2:**
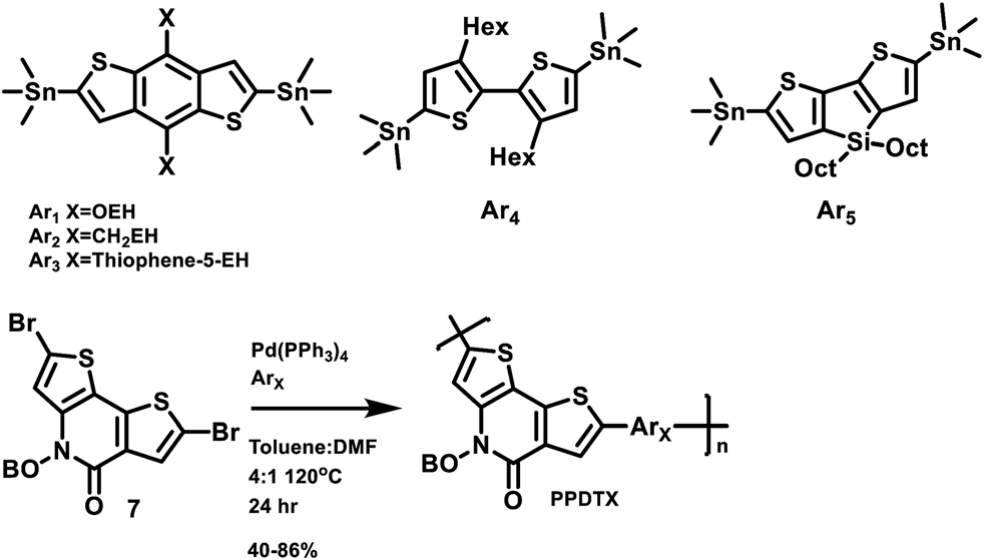
Synthetic route to **PPDT** series polymers.

The structures of the polymers synthesized here are shown in Fig. 1. The key monomer, *N*-alkyl 2-pyridone dithiophene (**PDT**), was synthesized in 5 steps as shown in Scheme 1 *via* the alkylation of Boc-protected 3-aminothiophene **1** followed by deprotection of the resulting alkylated amine **2** with trifluoroacetic acid. The resulting deprotected amine **3** was not isolated due to the known oxidative instability of electron rich thiophene amines, and was instead directly reacted with 2-bromo-3-thiophene carboxylic acid chloride **4** to produce the precyclized unit **5**. This was finally cyclized through an intramolecular Pd-catalyzed direct arylation to afford the **PDT** unit **6**. Bromination using NBS afforded the final monomer **7** (Scheme 1). These monomers were purified by column chromatography and recrystallized from hexane. Di-tin co-monomers were each synthesized according to the literature procedures.^42–46^ The Stille polycondensation shown in Scheme 2 with one of five different di-stannyl co-monomers generates the corresponding polymers in good yields. ^1^H-NMR and elemental analysis were used to characterize the structures of the polymers, which are all consistent with those proposed (Table S1†). The dispersity (Đ) and molecular weights of these polymers were measured by using gel permeation chromatography (GPC) with polystyrene as the standard. The results are shown in Table 1. These polymers are generally thermally stable until about 450 °C. Detailed thermal measurement data are shown in the supporting materials (Fig. S2†).

**Table 1:** Summary of **PPDT** series physical properties

Polymer	*M*_n_ (kDalton)	Đ	*λ*_onset_ ^*a*^ (nm)	*λ*_max_ ^*a*^ (nm)	HOMO (eV)	LUMO (eV)	*E* elec g (eV)	*E* opt g ^*c*^ (eV)
**PPDT1**	49.6	1.8	620	570, 527	–5.27	–2.68	2.59	2.00
**PPDT2**	41.8	1.8	630	573, 530	–5.47	–2.68	2.79	1.97
**PPDT3**	32.4	1.7	650	579, 536	–5.36	–2.83	2.53	1.90
**PPDT4**	30.4	1.5	610	500	–5.59	–2.77	2.82	2.03
**PPDT5**	12.7	2.4	680	574, 625^*b*^	–5.39	–2.87	2.52	1.82

^*a*^Taken thin film spectra.

^*b*^Shoulder.

^*c*^ *E* opt g = 1240/*λ*_onset_.

### Optical properties

**Fig. 2:**
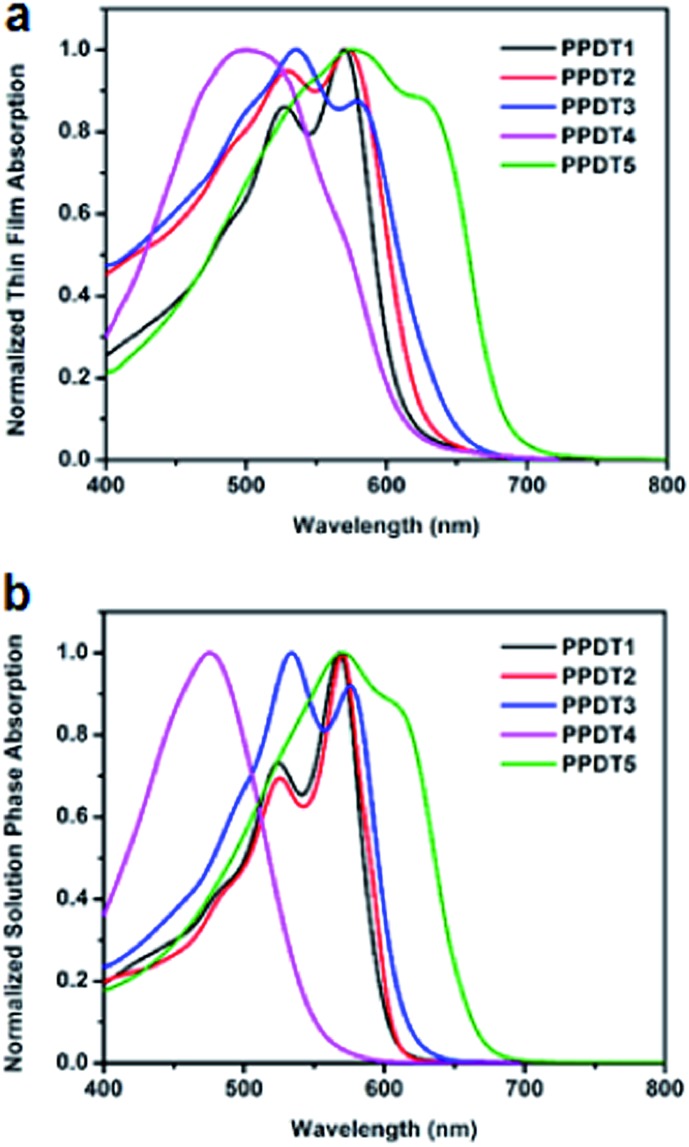
UV-vis absorption spectra of **PPDT** polymers. (a) Thin Film Absorption (b) solution phase absorption.

The UV-vis absorption spectra of the polymer films are shown in Fig. 2a. The **PPDT1–3** polymers have similar optical spectra due to the similar backbones, and only slight red-shifts are observed in the absorption peaks of **PPDT3**, possibly due to increased conjugation with the additional thiophene moieties in the direction perpendicular to the conjugated backbone. The absorption peak of **PPDT4** is significantly blue-shifted with much less pronounced vibronic features than other polymers, and resembles the absorption spectrum of P3HT at room temperature, which is known to have a disordered structure unless special care is taken during fabrication.^28^ Furthermore, **PPDT4** has a greater redshift between the solution and film state, which is consistent with a disordered structure. This disordered structure with attenuated vibronic features is most likely due to the reduction of the conjugation length along the polymer backbone caused by rotation about the C–C bond between the two thiophene rings with the low rotational barrier as well as the steric repulsion of the side chains favoring less backbone planarity, and hence lowered crystallinity. In contrast, the absorption of **PPDT5** is red-shifted significantly, likely due to the electron-rich silanyl substituent, thereby enhancing the charge transfer character in each repeating unit.^1^ The solution-phase spectra (Fig. 2b) exhibit features very similar to those in the thin films, suggesting significant polymer aggregation in solution. However, overall the solution phase polymer spectra are blue-shifted and become spectrally narrower, indicating that the local conformations become more uniform while the statistically averaged conjugation length is reduced in the solution phase.^28^

Polymer bandgaps are estimated from optical absorption spectra and by cyclic voltammetry (CV) of thin films coated on a Pt wire against a Ag/Ag^+^ reference electrode in acetonitrile solution containing a 0.1 M tetrabutylammonium hexafluorophosphate electrolyte. Both oxidation and reduction features are evident in the CV and show the wide bandgap nature of this series compared to the usual high-efficiency polymers (Fig. S3†). All of the polymers exhibit relatively low HOMO energies except **PPDT1**. The LUMO energies all lie above 3.0 eV (Table 1).

### Device fabrication and properties

**Fig. 3:**
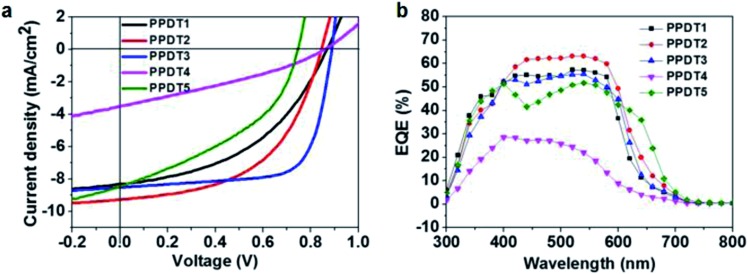
(a) Current density *vs.* voltage characteristics of optimized **PPDT** : PC_71_BM solar cells. (b) EQE curves for the **PPDT** : PC_71_BM solar cells in (a).

The photovoltaic performance of the polymers was measured in the following simple device structure: indium tin oxide (ITO)/poly(3,4-ethylenedioxythiophene): poly(styrenesulphonate) (PEDOT:PSS)/polymer : PC_71_BM (1 : 1.5 weight ratio)/Ca/Al. Fig. 3a shows the current density *vs.* voltage (*J*–*V*) characteristics of these devices under simulated AM 1.5 G illumination at 100 mW cm^–2^. The corresponding photovoltaic parameters are summarized in Table 2. Average PCE values from six identical devices are summarized in Table S2.† Thicknesses of the devices are approximately 100 nm. Except for **PPDT3**, all **PPDT** polymers are processed from chloroform solutions due to their poor solubility in chlorobenzene. Among all of the five polymers, the **PPDT3** : PC_71_BM device gives the highest performance with a short circuit current (*J*_sc_) at 8.50 mA cm^–2^, an open circuit voltage (*V*_oc_) at 0.89 V, a fill factor (FF) at 70.6% and a PCE of 5.33%. Also of note is the poor performance of **PPDT5**, which may be compromised by the lower *M*_n_ and higher dispersity compared to the other members of the series. Both of these factors are known to reduce PSC performance.^47,48^

**Table 2:** Photovoltaic parameters for **PPDT** : PC_71_BM solar cells^*a*^

Polymer	*V*_oc_ (V)	*J*_sc_ (mA cm^–2^)	FF (%)	PCE (%)
**PPDT1** ^a^	0.87	8.30	45.3	3.28
**PPDT2** ^a^	0.85	9.26	52.1	4.08
**PPDT3** ^b^	0.89	8.50	70.6	5.33
**PPDT4** ^a^	0.86	3.50	31.6	0.95
**PPDT5** ^a^	0.75	8.47	40.5	2.57

^*a*^Active layers are processed with ^a^chloroform and ^b^chlorobenzene, respectively.

The external quantum efficiencies (EQEs) of the present devices were measured to provide information on wavelength-dependent *J*_sc_ variations in these solar cells. As is shown in Fig. 3b, the **PPDT1**, **PPDT2** and **PPDT3** devices show similar EQE ranges from 300 nm to 650 nm while the **PPDT5** device exhibits an extended EQE range to 700 nm and the **PPDT4** device a narrower EQE range to 600 nm. These observations are in good agreement with the UV-vis absorption spectra for these polymers in Fig. 2. The integrated *J*_sc_ values for the five polymer blends are 8.52 mA cm^–2^, 9.67 mA cm^–2^, 8.39 mA cm^–2^, 3.56 mA cm^–2^ and 8.71 mA cm^–2^, respectively, which are within 5% of the *J*_sc_ values obtained from *J*–*V* measurements.

The hole mobilities of the pristine polymers were measured using the space-charge limited current (SCLC) method in the architecture: ITO/PEDOT:PSS/**PPDT** polymers/Al. Hole mobility values for the five polymers are 2.83 × 10^–4^ cm^2^ V^–1^ s^–1^ (**PPDT1**), 4.91 × 10^–4^ cm^2^ V^–1^ s^–1^ (**PPDT2**), 4.42 × 10^–4^ cm^2^ V^–1^ s^–1^ (**PPDT3**), 2.09 × 10^–5^ cm^2^ V^–1^ s^–1^ (**PPDT4**) and 4.40 × 10^–4^ cm^2^ V^–1^ s^–1^ (**PPDT5**), respectively. **PPDT4** shows a mobility value one order of magnitude smaller than that of the other 4 **PPDT** polymers, which is consistent with the much lower *J*_sc_ value for the **PPDT4** : PC_71_BM device.

**Fig. 4:**
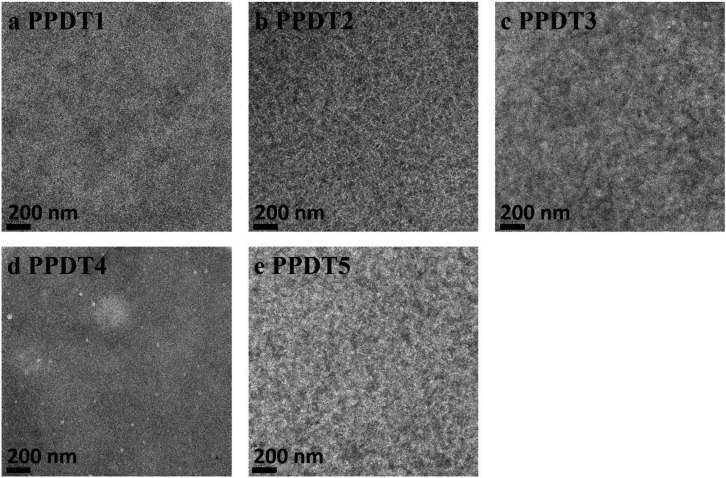
(a–e), TEM images of optimized **PPDT** : PC_71_BM devices. The scale bar in the TEM images is 200 nm.

Transmission electron microscopy (TEM) was used to investigate possible morphological differences in the active layers of these polymer : PC_71_BM devices (Fig. 4). **PPDT4** : PC_71_BM shows the finest phase separation, which could lead to severe bimolecular recombination due to the lack of bicontinuous charge transport channels. Dependence of *J*_sc_ on light intensity was measured to provide more insight into bimolecular recombination changes for the **PPDT4** device. Fig. S4† shows the logarithmic plots of *J*_sc_ *vs.* light intensity for **PPDT3** and **PPDT4**. The slope (s) of the graph of log(*J*_sc_) ∝ *s* log(*P*), is 0.89 for **PPDT4**. In contrast, for **PPDT3**, the best performing polymer in the series, this value is increased to 0.94, indicating decreased bimolecular recombination in **PPDT3** device compared to **PPDT4**, which is consistent with the lower hole mobility of **PPDT4**. This helps to explain why **PPDT4** achieves a PCE of only 0.95%. In addition, the other four polymer : PC_71_BM blends all show fibrillar microstructures with different domain sizes. **PPDT3** and **PPDT5** exhibit larger domain sizes than **PPDT1** and **PPDT2** as shown in Fig. 4.

### X-ray scattering data

**Fig. 5:**
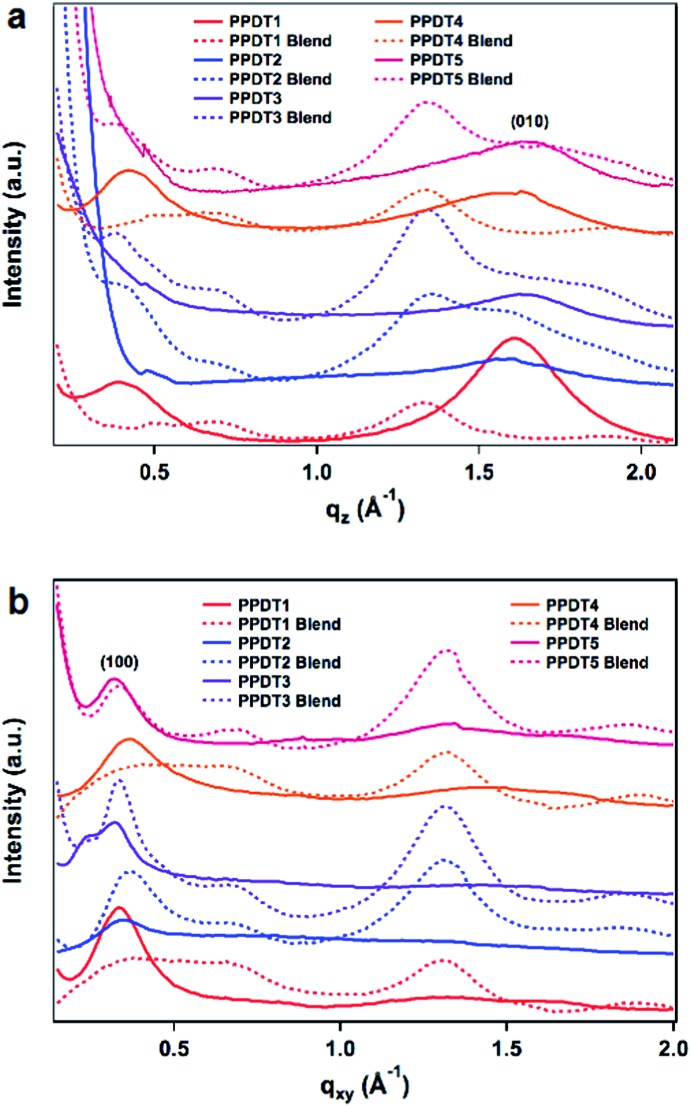
In plane (a) and out of plane (b) linecuts of polymer (solid lines) and **PPDT** : PCBM blends (dashed lines). The preferential face-on (100) and (010) polymer peaks are labeled for clarity. The large peak at *q* ≈ 1.3 in the blend films is attributed to PC_71_BM.

To further investigate morphology in this polymer series, grazing incidence wide-angle X-ray scattering (GIWAXS) was employed to probe the crystalline intermolecular interactions in the polymer films. All of the pristine polymers evidence preferential π-face-on polymer backbone orientation relative to the substrate at the interface as indicated by the out of plane (010) π–π stacking peak evident in all the neat films. The in-plane and out-of-plane linecuts are shown in Fig. 5 and the *d*-spacing and correlation lengths are summarized in Table 3. π–π stacking distances range from 3.86 to 4.08 Å, with the largest distance being exhibited by **PPDT4** – an expected result due to the aforementioned steric twist in the backbone and consistent with the reduced SCLC hole mobility relative to the other polymers. The in-plane (100) lamellar peaks exhibit stacking distances between 17.5 and 19.1 Å.

**Table 3:** GIWAXS-derived *d*-spacing and correlation length data calculated for pristine and blend **PPDT** films^*a*^

	*d*-Spacing (Å)	Correlation length (nm)		
	(100)	(010)	(100)	(010)
**PPDT1**	18.6	3.96	3.2	3.0
**PPDT1** blend	*	*	*	*
**PPDT2**	18.5	4.02	4.2	2.1
**PPDT2** blend	17.2	Broad	3.3	*
**PPDT3**	19.6	3.86	6.0	2.7
**PPDT3** blend	18.7	Broad	7.1	*
**PPDT4**	17.5	4.08	2.7	1.3
**PPDT4** blend	*	*	*	*
**PPDT5**	19.1	3.87	2.8	1.7
**PPDT5** blend	18.7	Broad	3.8	*

^a^* Peak not evident or Scherrer analysis could not be performed. Broad: too broad for analysis.

When blended with PC_71_BM (1 : 1.5 polymer : PC_71_BM weight ratio) it appears that the PCBM disrupts the polymer crystalline domains as the films become significantly more amorphous. Thus, **PPDT1** and **PPDT4** appear to lose all significant Bragg reflections, a result that is consistent with the TEM data and with their lower PCEs. **PPDT2**, **PPDT3**, and **PPDT5** all show a reduction in the (100) lamellar stacking distance, and have very broad Bragg peaks in the high *q* region, signifying less ordered π–π stacking that cannot be deconstructed into individual distances. Correlation lengths of the domains with periodic spacings were calculated *via* Scherrer analysis modified for a 2D detector utilizing the method outlined by Smilgies.^49^ The Scherrer analysis reveals that the crystalline domain sizes remain small with the largest neat domain being 6.0 nm in **PPDT3**. **PPDT1** and **PPDT4** have both the (100) and (010) peaks disappear completely in the blend film. In **PPDT2**, **PPDT3** and **PPDT5** blend films both peaks are maintained, but the (010) is too broad for Scherrer analysis. **PPDT3**, which has the highest fill factor and efficiency, demonstrates (100) domain sizes almost twice the size of the other polymers and maintains the (010) peak. The smaller crystalline domains for the other polymers in blend films are consistent with why the fill factors remain a limiting variable in the device efficiencies for the rest of the series as they lack the segregated pathways for holes and electrons to reduce bimolecular recombination.^1,7^

## Conclusions

A new polymer series, **PPDT** based on the pyridinone-dithiophene unit was designed, synthesized, and characterized in detail. Bulk heterojunction solar cells fabricated with these materials achieve PCEs of more than 5.3%. The **PPDT** series is also noteworthy for the high *V*_oc_s of nearly 0.9 eV, which are among the highest achieved to date for single junction cells. Furthermore, these materials absorb in a different region of the solar spectrum *versus* state-of-the-art low bandgap materials, providing promising candidates for use in alterative PSC device architectures such as tandem or ternary cells.
