# Supplementary material for: Wide bandgap OPV polymers based on pyridinonedithiophene unit with efficiency >5%
Source: Chem Sci. 2015 Jun 4;6(8):4860–6. doi: 10.1039/c5sc01427a (PMC5664788; doi:10.1039/c5sc01427a)
Supplement: Supplementary file 1 [file SC-006-C5SC01427A-s001.pdf]

Electronic Supplementary Material (ESI) for Chemical Science.
This journal is © The Royal Society of Chemistry 2015

Electronic Supplementary information For:

Wide Bandgap OPV Polymers Based on Pyridinonedithiophene Unit with Efficiency >5%

Alexander M. Schneider,[a,†] Luyao Lu,[a,†] Eric Manley,[b,c] Tianyue Zheng,[a] Valerii Sharapov[a] Tao Xu,[a] Tobin J. Marks,[b] Lin X. Chen,*[b,c] and Luping Yu*[a]

†A.M.S., and L.L. contributed equally.

a.: Department of Chemistry and The James Franck Institute, The University of Chicago, 929 E 57th Street, Chicago, IL 60637

b.: Department of Chemistry and ANSER Center, Northwestern University, 2145 Sheridan Road, Evanston, IL 60208

c.: Chemical Sciences and Engineering Division, Argonne National Laboratory, 9700 S. Cass Ave., Lemont, IL 60439

* To whom correspondence should be addressed. Email: lupingyu@uchicago.edu, l-chen@northwestern.edu

Materials Characterization and Spectroscopy......2

Solar Cell Fabrication and Testing......2

GIWAXS Experimental Setup......2

Synthesis of Materials......2-4

General Synthetic Scheme for Polymers......4

Monomer Energy Level Characterization......4

Polymer TGA Traces......5

Polymer CV Curves......5

Light Intensity Study for PPDT3 and PPDT4......5

GIWAXS 2D Spectra for Pure Polymers and Polymer:PCBM Blends......6

Polymer ^{1}H-NMR Spectra......7-9

Polymer Elemental Analysis......10

Average Solar Cell Parameters for Different Systems......10

Materials characterization and spectroscopy. ^{1}H- and ^{13}C-NMR spectra were recorded on a Bruker DRX-500 spectrometer in $CDCl_3$. Mass spectra were obtained on a Bruker Ultraflextreme MALDI-TOF/TOF mass spectrometer using dithranol as a matrix. UV-vis spectra were taken on a Shimadzu UV-3600 spectrofluorometer in chloroform or drop-cast on glass slides. Photoluminescence spectra were taken on a Horiba FL-1039/40. Cyclic Voltammetry data was taken with an AUTOLAB/PG-STAT12 system using a three electrode system with a polymer-coated Pt working electrode, a Pt wire auxiliary electrode, and a Ag/$AgNO_3$ reference electrode in 0.1M $[^{n}Bu_4N]^{+}[PF_6]^{-}$ solution in degassed acetonitrile. TGA data was measured on a TA Q600 instrument using ca. 2mg of polymer in an Al pan heated to 550°C. Polymer Molecular weights were measured using Gel Permeation Chromatography (GPC) on a Waters Associates Liquid Chromatography instrument equipped with a Waters 510 HPLC pump, a Waters 410 differential refractometer, and a Waters 486 tunable absorbance detector using chloroform as the eluent and polystyrene as the standard. Elemental Analysis was performed by Atlantic MicroLab, Inc. in Norcross, GA.

Solar cell fabrication and testing. Polymers (10 mg/mL) and $PC_{71}BM$ (15 mg/mL) were dissolved in chlorobenzene/chloroform and 1, 8-diodooctane co-solvent (97:3,v/v) and stirred on hot plate at 100 ^{o}C overnight. ITO coated glass substrates (Thin Film Devices) were cleaned with water, acetone and isopropyl alcohol under sonication for 15 min. They were then exposed to ultraviolet ozone irradiation for 1 hour. PEDOT:PSS (Clevios™ P VP AI 4083) layer (~ 40 nm)was then spin-coated and dried at 80 ^{o}C for 30 mins. Polymer:$PC_{71}BM$ solutions were then spin coated in glove box. After that, metal cathodes were thermal evaporated. The as prepared solar cells were measured under 1-sun, AM 1.5G irradiation.

GIWAXS Experimental Setup. Grazing incidence wide-angle x-ray scattering measurements were performed at Beamline 8-ID-E of the Advanced Photon Source at Argonne National Laboratory. The photon energy is 7.35 keV (λ = 1.6868 Å) and data were collected on a Pilatus 1M pixel array detector at a sample-detector distance of 204 mm. All films were prepared with 10mg/ml of polymer in 97:3 v/v CF/DIO solution. Blend films had a 1:1.5 Polymer to $PC_{71}BM$ ratio. All films were spun on clean Si at 1500 rpm for 1 minute. Spectra were collected at an incidence angle of 0.2°; the films were exposure for 25 seconds. To account for the gaps in the detector array, two images were taken per sample, one with the detector in the standard position and the other translated 23 mm down to fill the gap, the two images are then merged. Some of the gap fills were unsatisfactory and that section of those line cuts have been omitted for clarity.

1D line cuts were taken of the 2D scattering spectra in the in-plane and out-of-plane direction using the gixsgui software package developed by the beamline scientists at APS 8-ID-E. To normalize for the differing exposure times and air scatter, the line cuts were background subtracted utilizing an exponential fit. The background-subtracted peaks were fit using the multipeak fit function in igor pro. Scherrer analysis was performed utilizing the previously mentioned method by Smiglies to account for instrumental broadening and detection limits. The values presented represent a lower limit for correlation length, as the Scherrer analysis does not account for broadening due to defects in the crystallites.

Synthesis of materials. Detailed synthetic schemes of polymers and monomer precursors can be below. All reagents were purchased through commercial suppliers and used directly unless otherwise specified. Toluene and THF were dried over sodium and distilled prior to use.

***tert*-butyl (2-butyloctyl)(thiophen-3-yl)carbamate** (**2**)

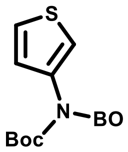

tert-Butyl (3-thienyl)carbamate (**1**, 5.0g, 25.1mmol) was dissolved in 250mL DMF and cooled to 0°C. NaH (2.0g 60% dispersion in mineral oil, 50mmol) was added and the solution was allowed to rise to room temperature. 5-(bromomethyl)-undecane (9mL, 37.5mmol) was added and the solution was allowed to stir overnight. The mixture was concentrated and then poured into water, followed by extraction with ethyl acetate 3x, the organic layers were combined, washed with water 3x, washed with brine, then dried over Na_2SO_4. The resulting yellow oil was purified via column chromatography starting with hexane as eluent then changing to 9:1 Hexane:EtOAc resulting in a clear oil in 89% yield. ^{1}H-NMR ($CDCl_3$, 500hz): δ 7.18 (1H, dd, j=10,7) 7.03,6.98 (2H, br d) 3.57 (2H, d, j=15) 1.60 (1H, m) 1.47 (9H, s) 1.22 (16H, m) 8.86 (6H, m) ^{13}C-NMR ($CDCl_3$, 500hz): δ 154.6, 141.1, 125.5, 123.7, 115.5, 80.3, 53.4, 36.6, 31.9, 31.4, 31.1, 29.8, 28.6, 28.4, 26.3, 23.2, 22.7, 14.2, 14.1 MALDI: 312 m/z (**2** – isobutylene + H)

***N*-(2-ethylhexyl)- 3-Thiophenamine** (**3**)

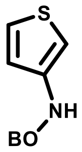

(8.2g, 22.3 mmol) was dissolved in 220mL DCM and cooled to 0°C. 15mL TFA was added and the solution was allowed to warm to room temperature and stir for 24 hours. Reaction turned pink upon stirring. Poured into water, washed with sodium bicarbonate, washed with brine, dried over Na_2SO_4 to afford a reddish-purple oil. Used without further purification or characterization.

2-Bromo-3-Thiophenecarbonyl chloride (**4**)

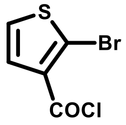

2-bromo-3-thiophenecarboxylic acid (7.0g, 33.6mmol) was added to a flame-dried flask equipped with a condenser. 140mL distilled toluene, 15mL $SOCl_2$ and ~1mL DMF were added and the solution was heated to reflux, after which it dissolved. This was allowed to reflux overnight. The solution was then cooled and the solvents were removed via rotovap. More toluene was added and this was also removed. The light brown solid was dried and then directly used in the next reaction with no further purification or characterization.

2-bromo-N-(2-butyloctyl)-N-(thiophen-3-yl)thiophene-3-carboxamide (**5**)

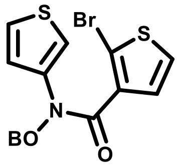

600mg NaH (60% in mineral oil, 15.0mmol) was dispersed in 60mL distilled toluene at 0°C. **3** (1.3g, 5 mmol) was added and the solution was allowed to rise to room temperature and stirred for 30 minutes. The solution was cooled back down to 0°C and a solution of **4** (1.1g, 5mmol) dissolved in toluene was added dropwise. The mixture was allowed to stir overnight. Solution was allowed to cool, then poured into water. The organic layer was washed with water twice, then brine, then dried over Na_2SO_4. Toluene was removed, and the product was purified by column chromatography (30% EtOAc:70% Hexane) 1.5g produced (65% yield) ^{1}H-NMR ($CDCl_3$, 500hz): δ 7 7.13 (1H, br s) 7.00 (1H, br s) 6.86 (1H, br s) 6.78 (1H, br s) 3.81 (2H, d j=12) 1.62 (1H, m) 1.2-1.4 (16H, m) 0.87 (6H, m) ^{13}C-NMR ($CDCl_3$, 500hz): δ 165.3, 140.6, 138.3, 127.0, 126.2, 125.6, 125.1, 119.8, 111.8, 52.5, 36.2, 31.9, 31.3, 31.0, 29.7, 28.6, 26.3, 23.1, 22.7, 14.2, 14.1 MALDI: 456 (90%) 458 (100%) m/z (**5** + H)

4-(2-butyloctyl)dithieno[3,2-b:2',3'-d]pyridin-5(4H)-one (**6**)

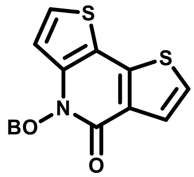

Combine **5** (1.5g, 3.3mmol) with $Pd(OAc)_2$ (74mg, 0.33mmol), K_2CO_3 (450mg, 3.3mmol) pivalic acid (102mg, 1mmol) and 33mL degassed DMA in a round bottom flask equipped with a condenser. The setup was subjected to 3 cycles of pump/backfill with Ar and then heated to 110 °C overnight. Cool, pour into water, extract with EtOAc, wash with water, brine, dry over Na_2SO_4. Produced 1.1g (90% yield) of red oil with blue fluorescence. ^{1}H-NMR ($CDCl_3$, 500hz): δ 7.70 (1H, d, j=11) 7.44 (1H, d, j=11) 7.23 (1H, d, j=11) 7.08 (1H, d, j=11) 4.22 (2H, br s) 1.99 (1H, m) 1.2-1.4 (16H, m) 0.86 (6H, m) ^{13}C-NMR ($CDCl_3$, 500hz): δ 158.7, 141.3,

140.4, 128.6, 126.4, 125.5, 122.5, 117.4, 114.1, 49.0, 37.1, 31.7, 31.4, 31.2, 29.5, 28.7, 26.5, 23.0, 22.5, 14.0, 14.0 MALDI: 376 m/z (**6** + H)

2,7-dibromo-4-(2-butyloctyl)dithieno[3,2-b:2',3'-d]pyridin-5(4H)-one (**7**)

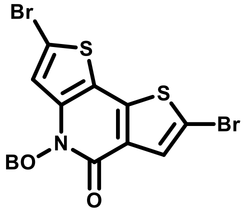

Dissolve **6** (0.43g, 1.14mmol) in DMF (25mL) and add NBS (2g, 4.6mmol). Cover reaction vessel with foil to protect from ambient light and allow to stir overnight. Dissolve in EtOAc, wash with $Na_2S_2O_3$ solution, wash with water, wash with brine. Dry over Na_2SO_4. Dry thoroughly under high vacuum and then recrystallize from Hexane. 590mg dark yellow powder produced (97% yield). ^{1}H-NMR ($CDCl_3$, 500hz): 7.63 (1H, s) 7.05 (1H, s) 4.11 (2H, br s) 1.92 (1H, m) 1.2-1.4 (16H, m) 0.86 (6H, m) ^{13}C-NMR ($CDCl_3$, 500hz): δ 157.2, 141.6, 140.0, 128.9, 128.8, 120.6, 115.1, 114.3, 111.0, 49.3, 37.2, 31.8, 31.4, 31.1, 29.6, 28.7, 26.5, 23.1, 22.6, 14.1, 14.1 MALDI: 532 (50%) 534 (100%) 536 (50%) m/z (**7** + H)

General synthetic scheme for polymers. 0.1 mmol **7** was combined with 0.1 mmol of desired distannyl coupling partner and 0.005 mmol tetrakis(triphenylphosphino)palladium(0) in a flame-dried 10 mL round-bottom flask equipped with a condenser. The reaction vessel was evacuated and backfilled with Ar 3 times, and then 3.3mL of anhydrous toluene and 0.8mL of anhydrous DMF were added. The resulting solution was heated to 120°C for 24 hours. After cooling, the polymer was precipitated from methanol, after which it was washed in a Soxhlet extractor successively with methanol, acetone, and hexanes, then extracted with chloroform. The chloroform portion was filtered through celite, then concentrated and once more precipiated from methanol, after which it was centrifuged and dried to afford the final polymer.

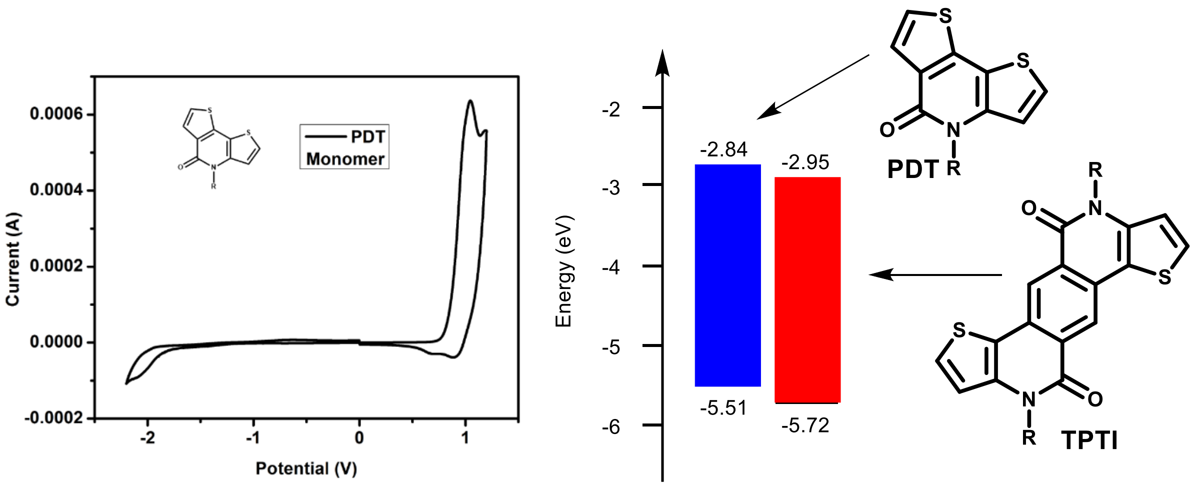

Figure S1. Monomer CV (left) and energy level comparison to TPTI (right).

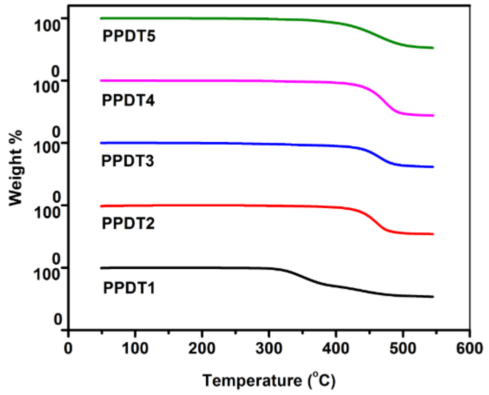

Figure S2. Polymer TGA Traces.

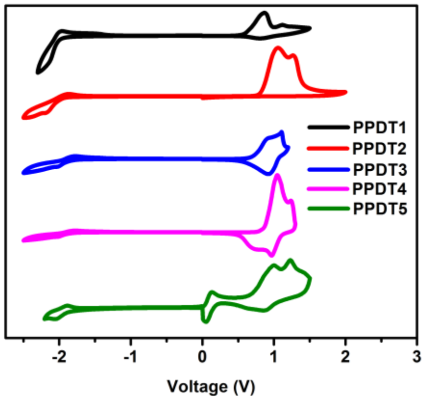

Figure S3. Polymer CV Curves.

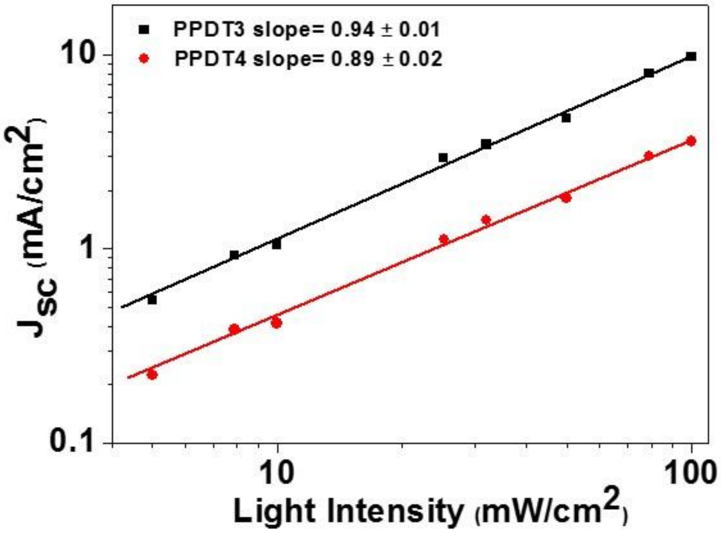

Figure S4. J_{sc} dependence on light Intensity measurements for PPDT3 and PPDT4 devices.

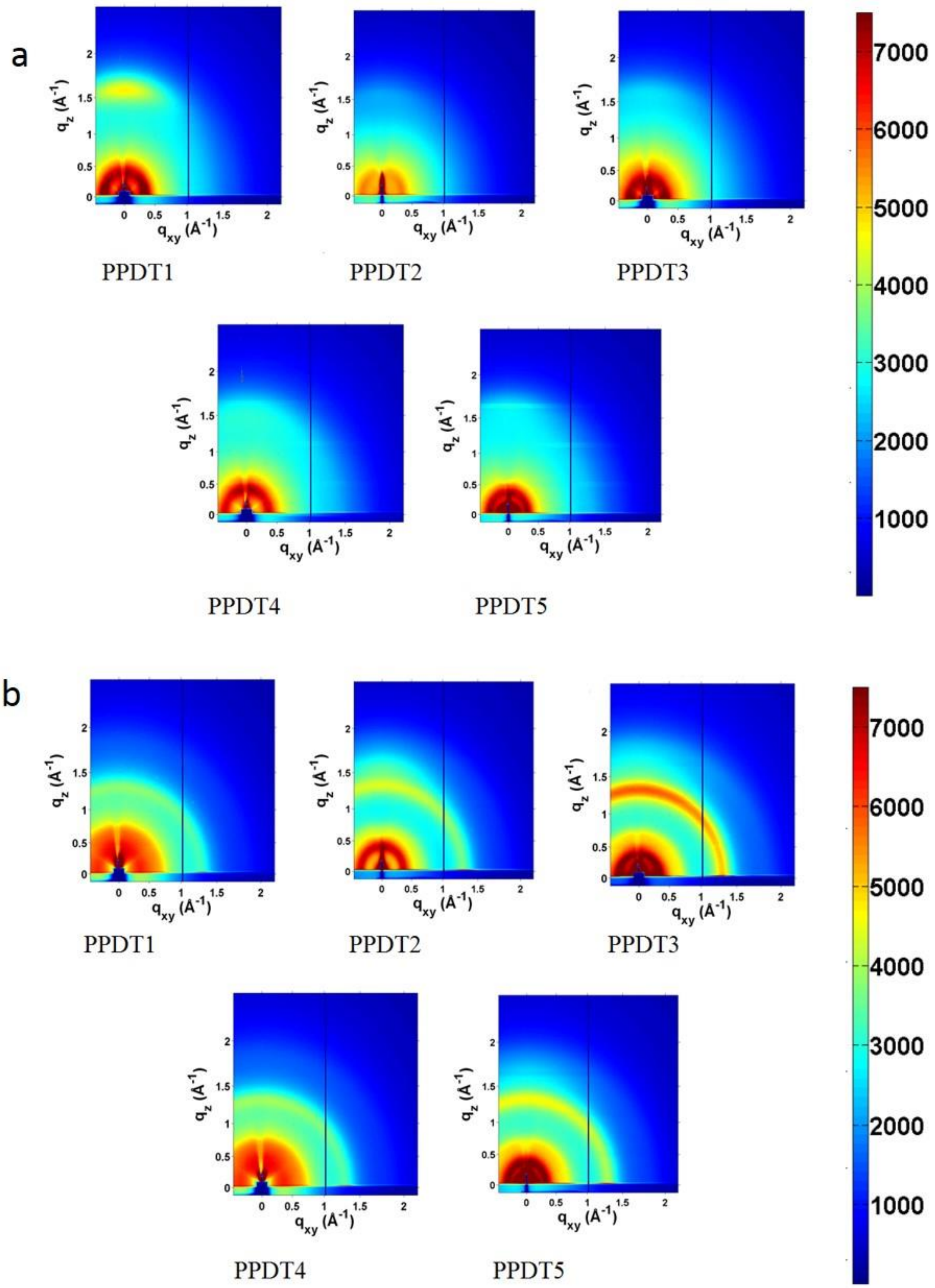

Figure S5. a, GIWAXS 2-D Scattering Images of Pure Polymers; b, GIWAXS 2-D Scattering Images of Polymer:PCBM blends.

Polymer ^{1}H-NMR

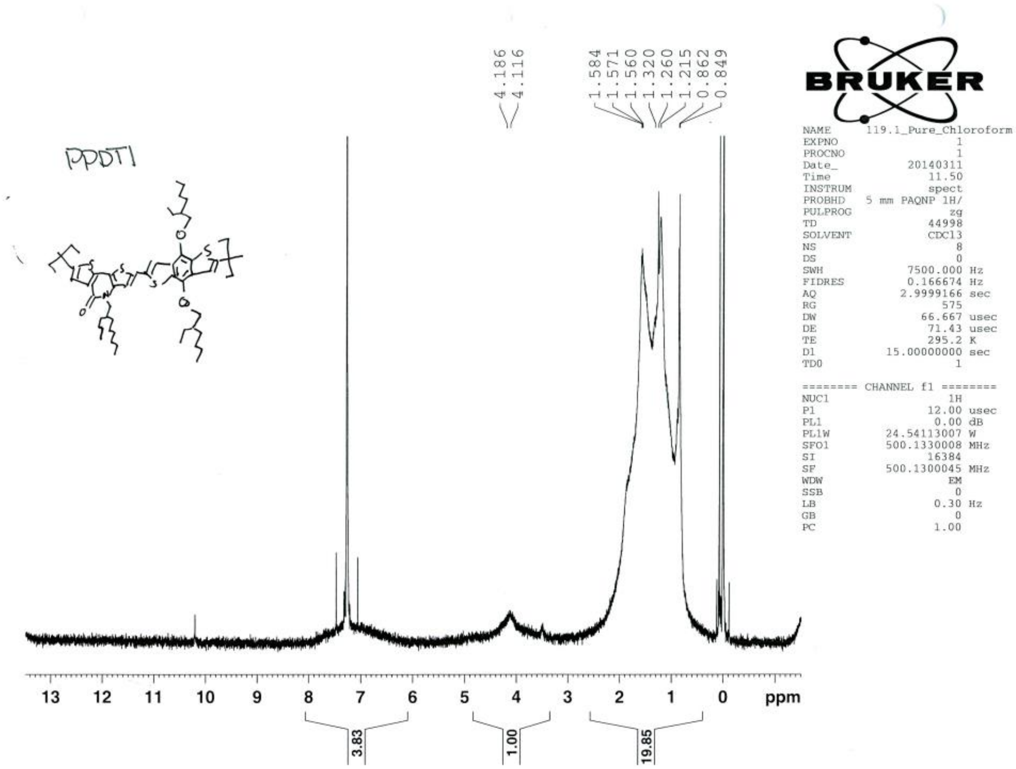

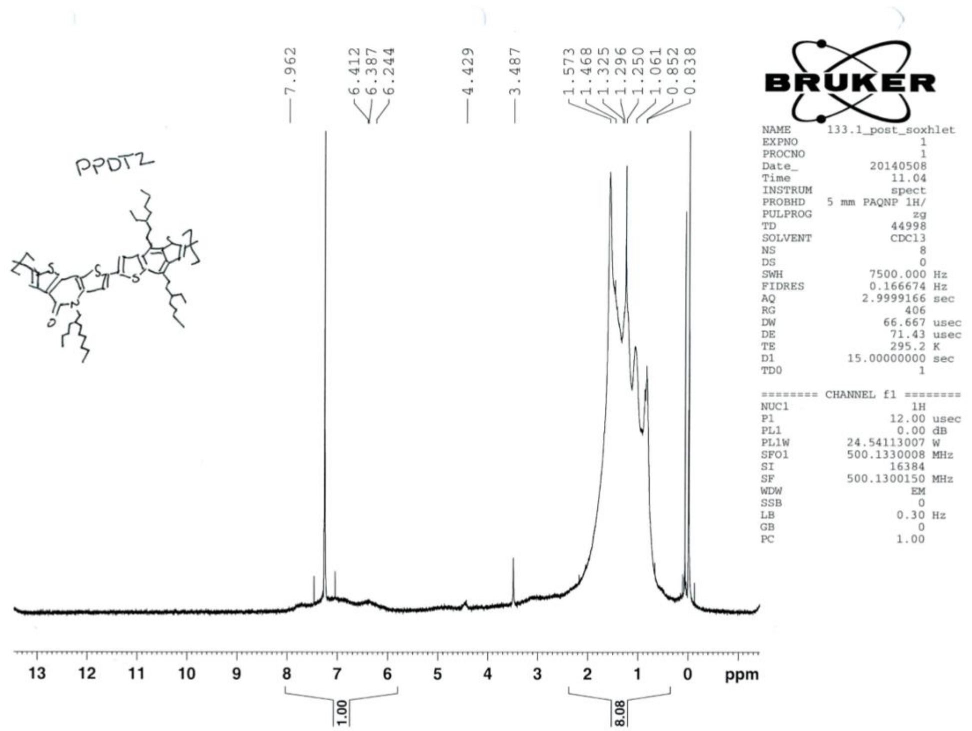
PPDT2
7.962
6.412
6.387
6.244
4.429
3.487
1.573
1.468
1.325
1.296
1.250
1.061
0.852
0.838
BRUKER
NAME 133.1_post_soxhlet
EXPNO 1
PROCNO 1
Date_ 20140508
Time 11.04
INSTRUM spect
PROBHD 5 mm PAQNP 1H/
PULPROG zg
TD 44998
SOLVENT CDCl3
NS 8
DS 0
SWH 7500.000 Hz
FIDRES 0.166674 Hz
AQ 2.9999166 sec
RG 406
DW 66.667 usec
DE 71.43 usec
TE 295.2 K
D1 15.00000000 sec
TD0 1
======== CHANNEL f1 ========
NUC1 1H
P1 12.00 usec
PL1 0.00 dB
PL1W 24.54113007 W
SFO1 500.1330008 MHz
SI 16384
SF 500.1300150 MHz
WDW EM
SSB 0
LB 0.30 Hz
GB 0
PC 1.00
13 12 11 10 9 8 7 6 5 4 3 2 1 0 ppm
1.00
8.08

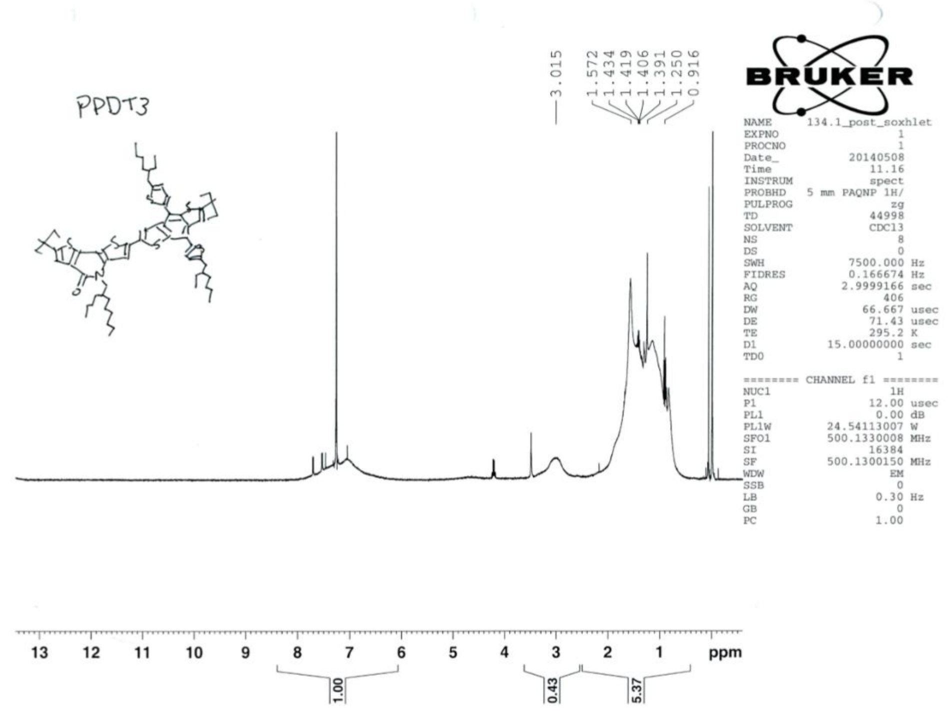
PPDT3
3.015
1.572
1.434
1.419
1.406
1.391
1.250
0.916
BRUKER
NAME 134.1_post_soxhlet
EXPNO 1
PROCNO 1
Date_ 20140508
Time 11.16
INSTRUM spect
PROBHD 5 mm PAQNP 1H/
PULPROG zg
TD 44998
SOLVENT CDCl3
NS 8
DS 0
SWH 7500.000 Hz
FIDRES 0.166674 Hz
AQ 2.9999166 sec
RG 406
DW 66.667 usec
DE 71.43 usec
TE 295.2 K
D1 15.00000000 sec
TD0 1
======== CHANNEL f1 ========
NUC1 1H
P1 12.00 usec
PL1 0.00 dB
PL1W 24.54113007 W
SFO1 500.1330008 MHz
SI 16384
SF 500.1300150 MHz
WDW EM
SSB 0
LB 0.30 Hz
GB 0
PC 1.00
13 12 11 10 9 8 7 6 5 4 3 2 1 ppm
1.00
0.43
5.37

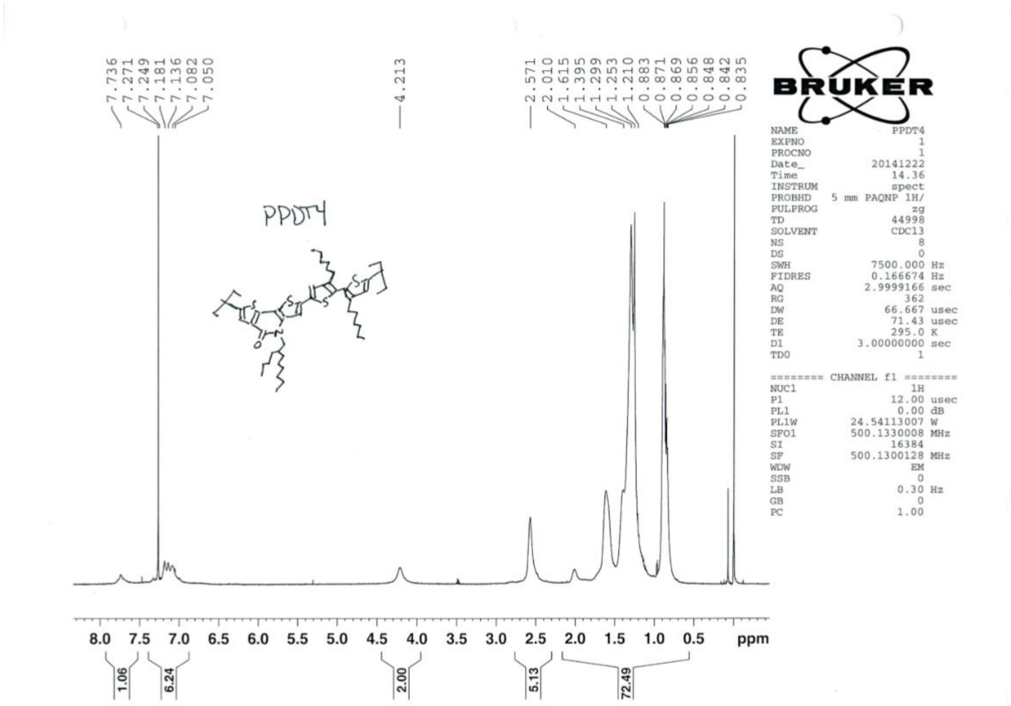
7.736
7.271
7.249
7.181
7.136
7.082
7.050
4.213
2.571
2.010
1.615
1.395
1.299
1.253
1.210
0.883
0.871
0.869
0.856
0.848
0.842
0.835
BRUKER
NAME PPDT4
EXPNO 1
PROCNO 1
Date_ 20141222
Time 14.36
INSTRUM spect
PROBHD 5 mm PAQNP 1H/
PULPROG zg
TD 44998
SOLVENT CDCl3
NS 8
DS 0
SWH 7500.000 Hz
FIDRES 0.166674 Hz
AQ 2.9999166 sec
RG 362
DW 66.667 usec
DE 71.43 usec
TE 295.0 K
D1 3.00000000 sec
TD0 1
======== CHANNEL f1 ========
NUC1 1H
P1 12.00 usec
PL1 0.00 dB
PL1W 24.54113007 W
SFO1 500.1330008 MHz
SI 16384
SF 500.1300128 MHz
WDW EM
SSB 0
LB 0.30 Hz
GB 0
PC 1.00
PPDT4
8.0 7.5 7.0 6.5 6.0 5.5 5.0 4.5 4.0 3.5 3.0 2.5 2.0 1.5 1.0 0.5 ppm
1.06
6.24
2.00
5.13
72.49

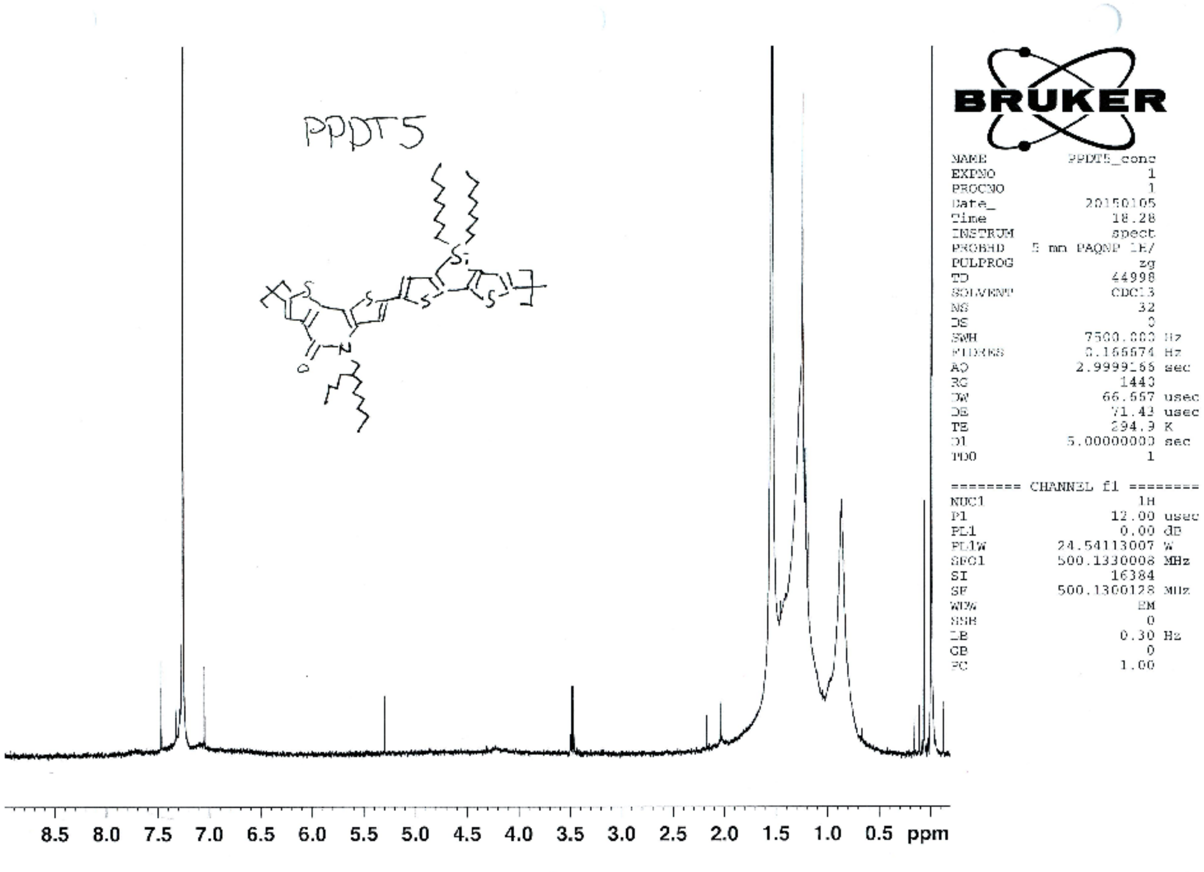
BRUKER
NAME PPDT5_conc
EXPNO 1
PROCNO 1
Date_ 20150105
Time 18.28
INSTRUM spect
PROBHD 5 mm PAQNP 1H/
PULPROG zg
TD 44998
SOLVENT CDCl3
NS 32
DS 0
SWH 7500.000 Hz
FIDRES 0.166674 Hz
AQ 2.9999166 sec
RG 1443
DW 66.667 usec
DE 71.43 usec
TE 294.9 K
D1 5.00000000 sec
TD0 1
======== CHANNEL f1 ========
NUC1 1H
P1 12.00 usec
PL1 0.00 dB
PL1W 24.54113007 W
SFO1 500.1330008 MHz
SI 16384
SF 500.1300128 MHz
WDW EM
SSB 0
LB 0.30 Hz
GB 0
PC 1.00
PPDT5
8.5 8.0 7.5 7.0 6.5 6.0 5.5 5.0 4.5 4.0 3.5 3.0 2.5 2.0 1.5 1.0 0.5 ppm

Table S1. Polymer Elemental Analysis.

Polymer	Calc C	Found C	Calc H	Found H	Calc S	Found S
**PPDT1**	68.98	65.60	7.77	7.78	15.67	13.33
**PPDT2**	72.27	71.72	8.29	8.23	15.75	15.23
**PPDT3**	69.50	68.37	7.11	7.17	20.24	19.55
**PPDT4**	69.74	69.44	7.85	7.83	18.16	18.05
**PPDT5**	68.39	67.09	8.03	7.98	16.23	15.81

Table S2. Summary of average J_{sc}, V_{oc}, *FF* and *PCE* values from six devices for each PPDT:PCBM system.

Polymer	V_{oc} (V)	J_{sc} (mA/cm^2)	*FF* (%)	*PCE* (%)
**PPDT1**	0.87±0.01	8.22±0.08	44.9±0.40	3.20±0.08
**PPDT2**	0.85±0.01	9.10±0.15	52.5±0.31	4.04±0.12
**PPDT3**	0.89±0.01	8.33±0.12	70.3±0.26	5.19±0.14
**PPDT4**	0.86±0.01	3.27±0.21	31.0±0.36	0.86±0.08
**PPDT5**	0.75±0.01	8.05±0.24	40.5±0.23	2.43±0.09
